# Seizure Characteristics and Background Amplitude-Integrated Electroencephalography Activity in Neonatal Rats Subjected to Hypoxia–Ischemia

**DOI:** 10.3389/fped.2022.837909

**Published:** 2022-04-07

**Authors:** Xiaowei Sun, Fenqin Xue, Jialin Wen, Limin Gao, Yang Li, Qianqian Jiang, Lijun Yang, Hong Cui

**Affiliations:** ^1^Department of Pediatrics, Beijing Friendship Hospital, Capital Medical University, Beijing, China; ^2^Core Facility Center, Capital Medical University, Beijing, China

**Keywords:** seizures, electroencephalography (EEG), hypoxia, ischemia, premature, electrode

## Abstract

**Objective:**

Perinatal hypoxic–ischemic encephalopathy (HIE) is a major cause of epilepsy and chronic neurologic morbidity in premature infants. This study aimed to investigate the characteristics of acute seizures and the pattern of background activity on amplitude-integrated electroencephalography (aEEG) in neonatal rats with HIE.

**Methods:**

Hypoxia–ischemia (HI) was induced in postnatal day (P) 3 neonatal rats (n = 12) by ligation of the left carotid artery and exposure to airtight hypoxia for 2 h. Data regarding seizure type, frequency, and duration and those related to neurobehavioral development were collected, and the integrated power of background EEG was analyzed to evaluate the effect of HI.

**Results:**

All neonatal rats in the HI group experienced frequent seizures during hypoxia, and 83.3% of rats (10/12) experienced seizures immediately after hypoxia. Seizure frequency and duration gradually decreased with increasing age. The mortality rate of the HI group was 8.33% (1/12); 120 h after HI induction, only 27.3% (3/11) of pups had low-frequency and short-duration electrographic seizures, respectively. HI rats, which presented seizure activities 96 h after HI insult, exhibited an increase in righting reflex time and a decrease in forelimb grip reflex time. Background EEG was significantly inhibited during HI induction and immediately after hypoxia and gradually recovered 72 h after hypoxia.

**Conclusion:**

Seizures caused by HI brain damage in premature infants can be simulated in the P3 neonatal rat model.

## Introduction

Hypoxic–ischemic encephalopathy (HIE) is one of the most important causes of brain injury in preterm infants (1). HIE incidence is higher in preterm infants (4–48 per 1,000 preterm newborns) than in term infants (1–8 per 1,000 live births), indicating that hypoxia–ischemia (HI) plays an important role in the pathophysiology of preterm brain injury (2–4). HI brain injury mainly damages the thalamus, basal ganglia, cortex, and white matter in full-term neonates (5). In preterm infants, HI influences oligodendrocyte progenitor cell maturation, disrupts the myelination process, and leads to neuronal loss and diffuse damage to the periventricular white matter (6–8). Immature brains have been reported to show a different damage phenotype than mature brains (9, 10).

The Rice–Vannucci model of HI is the most widely well-established rodent model of neonatal HI since it was first established in P7 rats (10, 11). The Rice–Vannucci model has been modified to re-create different types and severities based on the age of injury, duration of hypoxia, and oxygen saturation (12). At postnatal day 3 (P3), rats are similar to very preterm infants regarding the stage of oligodendroglial maturation and axonal outgrowth, making them highly suitable for use in such studies (13, 14).

Effective tools for seizure detection include electroencephalography (EEG) and amplitude-integrated EEG (aEEG), in which data are derived from the raw EEG data after processing. The study and development of techniques that allow for the serial evaluation of aEEG data will help elucidate the effect of HIE on brain function and the risk of adverse neurological outcomes (15, 16). Due to technical limitations (e.g., small head size, unfixed skull, and the need for neonatal rats to live with their mothers for breastfeeding support) and other factors, a full description of EEG in a premature HIE model is also lacking. Video-EEG is the gold standard to identify seizures and to confirm clinical and subclinical seizures. However, video-EEG may lead to a missed diagnosis of clinical seizures without obvious physical signs in neonatal rats. Therefore, in addition to video, we also installed electromyography (EMG) electrodes in the neck muscles of neonatal rats to confirm whether there were behavioral seizures.

Animal models of HIE are critical to better understand the underlying mechanisms and pathogenesis of premature neonatal seizures and HI. Therefore, in this study, we aimed to describe the characteristics of acute seizures and background aEEG pattern from postnatal days 3 to 8 in a rat model of HIE using video-EEG and EMG recordings.

## Material and Results

### Animals

All Sprague–Dawley rats used in this study were obtained from Beijing Vital River Laboratory Animal Technology Co., Ltd. (Beijing, China) and were housed under controlled conditions (22°C ± 1°C, 55% relative humidity, 12-h light/dark cycle, and lights on at 8 a.m.) with access to water and food *ad libitum*. After the end of the daily recording, rats were returned to their home cages. All animal experiments were conducted after approval by the local ethics committee (Beijing Friendship Hospital Ethics Committee, Capital Medical University) and in compliance with the “Laboratory animals-General requirements for animal experiment” (GB/T 35823-2018, China).

### Electrode Implantation

**FIGURE 1:**
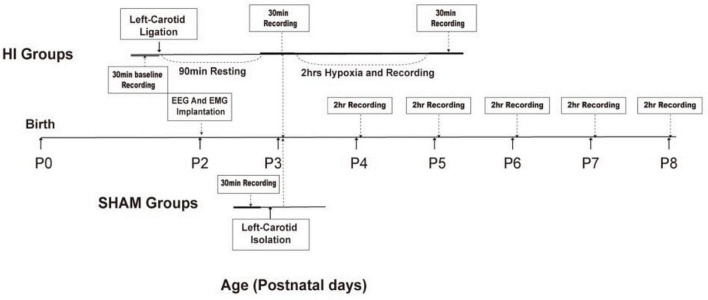
Schematic representation of the experimental design in HI and sham-control group rats. P, postnatal day; hrs, hours; HI, hypoxia–ischemia.

**FIGURE 2:**
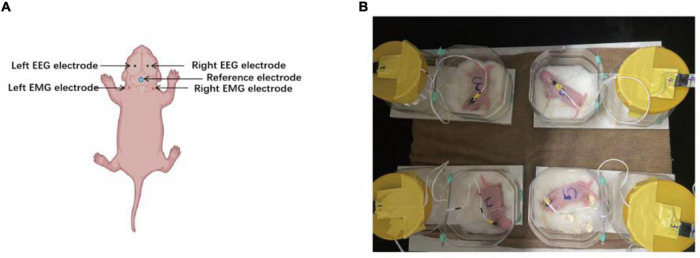
Location of electrodes placement and electroencephalography (EEG) monitoring. **(A)** Two silver electrodes were implanted bilaterally in the parietal cortex for EEG recording. The black dots are the location of the EEG electrodes. Two insulated stainless-steel wire electrodes were inserted bilaterally into the neck muscle for nuchal electromyography (EMG) recordings. The right dots are the location of the EEG electrodes. The blue-circled dots denote the location of reference electrode. **(B)** The signals from multiple receivers were collected and stored at a sampling rate of 1,000 Hz.

P2 rats (mean weight, 7.76 g; *n* = 20) were surgically implanted with two EEG electrodes, two EMG electrodes, and one reference electrode (Figure 1). Anesthesia was induced with 2% isoflurane and maintained with 1.5% isoflurane and 30% oxygen. After a midline vertical incision was made to expose the skull, forceps were used to remove any connective tissue, and the skull was dried for electrode placement. Electrodes were implanted bilaterally 2 mm before the lambda and 2 mm lateral from the midline sutures using a hand-held micro-drill. Moreover, a 1-mm hole was drilled 3 mm under the lambda to allow reference electrode implantation. Two insulated silver electrodes (0.008-inch outer diameter; A-M Systems, 131 Business Park Loop Sequim, WA, United States) were implanted bilaterally in the parietal cortex with a small amount of dental cement added to secure the electrodes to the skull. The depth of the EEG electrode from the surface of the skull to the cortex was 1 mm. This depth ensured that electrodes were located on the surface of the cortex to obtain a clear and good-quality signal. Two insulated stainless-steel wire electrodes were inserted into the neck muscle for nuchal EMG recordings, with the coated portion of the wire bent to follow the contour of the head. The five electrodes were connected to a 6-pin electrode pedestal, and the weight of the entire apparatus was approximately 220–230 mg. The bloodstains on the surface of the skull were cleaned, and the connection between the electrode and the skull was stabilized by dental cement. Figure 2A presents a schematic of electrode locations. After the procedure, pups were allowed to recover until the return of spontaneous movements, following which they were reunited with their mothers.

### Neonatal Rat Model of Hypoxic–Ischemic Brain Injury

After a 24-h recovery following electrode implantation, P3 neonatal rats (*n* = 20) were allocated to the HI (*n* = 12) and sham-control groups (*n* = 8) using the random Excel function. Thirty minutes of baseline activity was recorded preoperatively in both the HI and sham-control groups. After the induction of anesthesia with isoflurane, the neck was prepared and draped using standard sterile techniques. Subsequently, a small midline incision was made on the anterior neck, and the left carotid artery was isolated and double-ligated with a surgical suture. The artery was severed between ligations. All surgeries lasted no longer than 10 min. After the surgical procedure was completed, the pups remained with the dam in a warm cage to wake and recover for 90 min. Thereafter, the pups were separated and monitored for 30 min using video-EEG and EMG. They were subsequently placed in an airtight jar with 8% oxygen and balanced with 92% nitrogen for 2 h at 37°C. Video-EEG and EMG were continuously monitored during hypoxia (2 h) and recorded immediately for 30 min after hypoxia. In the sham-control group, the left carotid artery was isolated without ligation or hypoxic treatment. See Figure 1 for further details.

### Data Acquisition and Recording

Neonatal rats were separated from the dam and underwent video-EEG and EMG monitoring between P3 and P8. At P3, 30 min of baseline activity was recorded preoperatively in both the experimental and sham-control groups. Following ligation and before hypoxia, the experimental groups underwent 30 min of video-EEG and EMG monitoring and were continuously monitored during hypoxia for 2 h. The recording was continued for 30 min after hypoxia. All pups in the experimental and sham-control groups were separated from the dam and underwent 2 h of video-EEG and EMG monitoring every day between P4 and P8. After the recording, the pups were immediately returned to their mothers. A detailed workflow is shown in Figure 1. EEG and EMG signals were recorded by the Digital Headstage Processor (PLEXON, Dallas, TX, United States). The signals from multiple receivers (1 per animal) were collected at a sampling rate of 1,000 Hz and stored on a PC hard disk (Figure 2B).

### Seizure Definition

**FIGURE 3:**
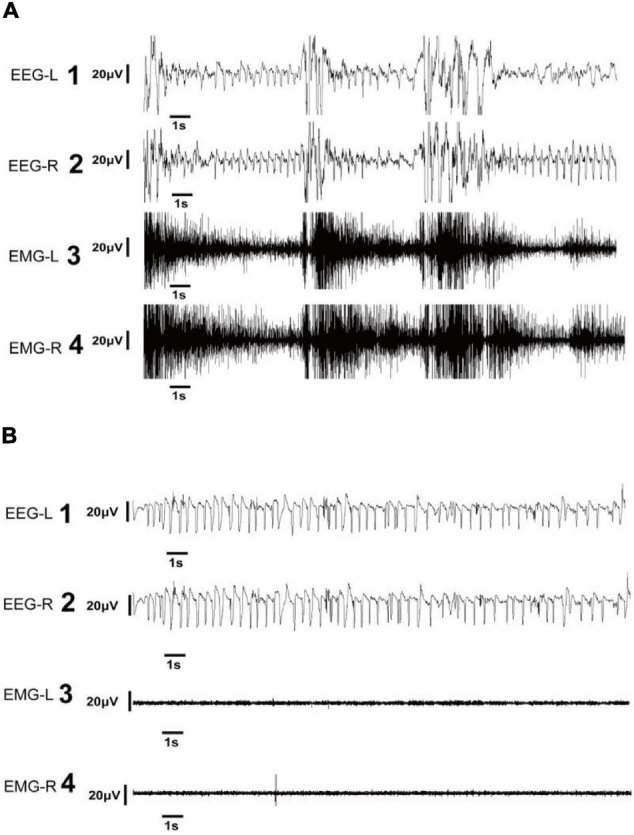
Representative electroencephalography (EEG) of electroclinical and electrographic seizures. **(A)** EEG and electromyography (EMG) in the left and right panels show electroclinical seizure activities. Electroclinical seizures were defined as typical events that met electrographic and behavioral criteria. The EEG trace showing the seizure with high-frequency, large-amplitude spike activity during tonic phase of seizure **(A1,2)** accompanied by high-amplitude EMG recordings **(A3,4)**. **(B)** EEG and EMG in the left and right panels show electrographic seizure activities. Electrographic seizures were defined as seizures observed in the EEG with high-frequency, large-amplitude spikes **(B1,2)**, and with an EMG pattern exhibiting a low magnitude **(B3,4)**. EEG-L, EEG in the left cortex; EEG-R, EEG in the right cortex; EMG-L, EMG in the left neck muscle; EMG-R, EMG in the right neck muscle.

Electroclinical seizures were defined based on the presence of the following for at least 10 s: 1) an EEG pattern that differed from the background in either amplitude, frequency, or both; and 2) an EMG pattern with a higher magnitude than the background EMG (Figure 3A). Rats were monitored for repetitive movements, such as extensor or flexor spasms of the body musculature, which are sometimes accompanied by forelimb pawing movements on the video. These spasms can be symmetrical or asymmetrical and are correlated with seizures on EEG and movement on EMG.

Electrographic seizures (EGS) were defined as seizures confirmed by EEG results that lasted at least 10 s and with an EMG pattern exhibiting the same magnitude as the background EMG (Figure 3B). Such seizures were not visibly correlated with behavioral movements on video and EMG.

EEG records were inspected by two independent observers (JW and YL) and analyzed by the first author (XS). All seizures were identified and checked by a professional electrophysiologist with extensive experience in animal EEG (FX).

### Assessment of Neurobehavioral Development

HI rats were divided into non-EGS and EGS groups according to whether EGS occurred 72 h following HI induction. Rats in the sham group were 6 days old. In the righting reflex test, pups were placed on a flat surface in the supine position, and the expected response was to turn over on the ventral surface, resting in the normal position with four feet on the ground, and the time was recorded as the average of three consecutive trials. In the forelimb grip strength test, the forelimbs were touched with a thin rod, and the time was recorded as the average of three consecutive trials.

### Statistical Analysis

NeuroExplorer version 5.0 (Nex Technologies) was used to open the stored data file and analyze the EEG and EMG data. Discrete Fourier transforms (DFTs) were performed to analyze EEG data in the frequency domain of between 0.5 and 40 Hz. Power spectral densities (PSDs) were obtained using 512 Hanning-window segments and normalized by 10 × log_10_(PSD). The actual power was normalized to investigate the integrated power. A one-way ANOVA was performed to evaluate the baseline integrated EEG power of the HI group in P3 after left carotid ligation, but before hypoxia, during hypoxia, and immediately after HI. The integrated power of background EEG of P4–P8 was compared between the sham and HI groups using the *t*-test. Data are presented as mean ± SEM. p < 0.05 was considered significant (*p < 0.05; ^**^p < 0.01; ^***^p < 0.001).

## Results

### Effect of Hypoxia–Ischemia on Electroencephalography Activity in Neonatal Rats

One rat died between 48 and 72 h after HI induction. The mortality rate of the HI experimental group was 8.3% (1/12). The mortality was 0 in the sham-control group. The rats of the sham group did not show any signs of seizure during any EEG recording. No seizure activity was detected on baseline EEG recordings in P3 rats subjected to HI (*n* = 12). Five of 12 pups (41.7%) developed short-duration EGS, and one pup (8.3%) experienced electroclinical seizures five times after left carotid artery ligation and before hypoxia. The electroclinical seizures consisted of head shaking as well as clonic and tonic limb movements. All pups (*n* = 12) experienced frequent EGS, and 10 of 12 rats (83.3%) developed frequent and long-duration electroclinical seizures during hypoxia. Within 30 min of the end of hypoxia, 10 of 12 (83.3%) rats experienced EGS, and some rats (66.7%; 8/12) also developed electroclinical seizures. Most pups (75%; 9/12) continued to have EGS 24 h after HI induction. Although the number of seizures was lower at 24 h than during HI induction, the seizures were still long. Half of the rats (6/12) also experienced low-frequency, long-duration electroclinical seizures. Five and seven of the 12 pups experienced electrographic and electroclinical seizures 48 h after HI induction, respectively. Seventy-two hours after the initial insult, 54.5% of pups (6/11) continued to exhibit EGS, and 45.5% (5/11) experienced electroclinical seizures. The seizure frequency and total seizure duration of these rats during the 72 h following HI induction were lower than those during and immediately following the hypoxic period. At 96 and 120 h after HI induction, no pups had electroclinical seizures, and only a few pups had low-frequency and short-duration EGS. More detailed information is presented in Table 1.

**TABLE 1:** Effects of hypoxia-ischemia on seizures in neonatal rats.

	No. pups total seizure	No. pups EGS	No. EGS	Total duration of EGS (min)	No. pups ECS	No. ECS	Total duration of ECS (min)
Baseline (30 min)	0/12	0/12	—	—	0/12	—	—
After carotid ligation before hypoxia (30 min)	5/12	5/12	2.2 ± 1.3	3.0 ± 2.2	1/12	5	13.1
During HI (120 min)	12/12	12/12	10.2 ± 12.5	4.0 ± 3.5	10/12	8.2 ± 6.7	8.2 ± 9.2
Immediately after HI (30 min)	10/12	10/12	2.9 ± 3.2	1.5 ± 2.0	8/12	1.3 ± 1.4	1.2 ± 2.1
24 h after HI (120 min)	9/12	9/12	7.4 ± 8.0	8.0 ± 10.0	6/12	2.5 ± 1.6	9.0 ± 11.7
48 h after HI (120 min)	7/12	5/12	4.3 ± 1.5	7.1 ± 1.7	7/12	4.2 ± 2.0	5.2 ± 2.7
72 h after HI (120 min)	6/11	6/11	2.0 ± 1.4	3.3 ± 2.4	5/11	2.3 ± 1.0	8.2 ± 13.1
96 h after HI (120 min)	4/11	4/11	1.3 ± 0.6	0.8 ± 0.5	0/11	—	—
120 h after HI (120 min)	3/11	3/11	1.0 ± 0	0.5 ± 0.3	0/11	—	—

*“No. pups total seizure” refers to the total number of seizures (including EGS and ECS) in rats recorded over a period of time. One rat died between 48 and 72 h after HI, and no other rat among 12 pups prepared died in this experiment. Data are shown as the mean values ± SD, n = 12.*

*HI, hypoxia–ischemia; EGS, electrographic seizure; ECS, electroclinical seizure.*

### Assessment of Neurobehavioral Development

**FIGURE 4:**
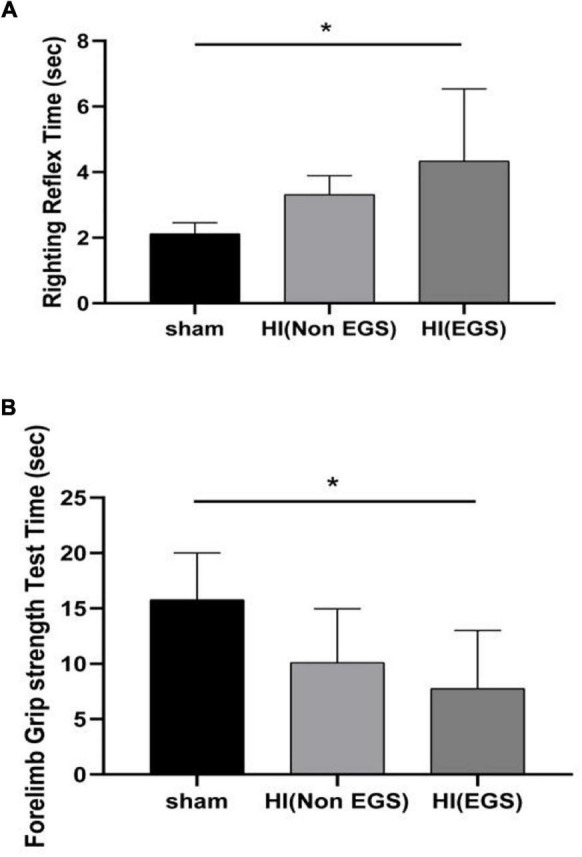
Neurobehavioral development evaluation of rats. Hypoxia–ischemia (HI) rats were divided into HI (Non-EGS) and HI (EGS), according to whether the electrographic seizures (EGS) occurred at 72 h following HI induction. The day age of the sham group rats is postnatal day 6. **(A)** Righting reflex test. **(B)** Forelimb grip strength test. *p < 0.05.

Rats in the HI (EGS) group after 72 h of HI induction exhibited significantly increased righting reflex time (Figure 4A) and significantly decreased forelimb grip time (Figure 4B) as compared with P6 rats aged the same in the sham group.

### Background Electroencephalography Activity

**FIGURE 5:**
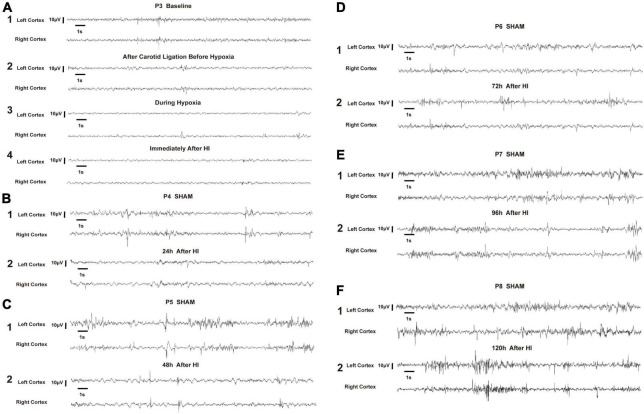
Representative electroencephalography (EEG) of hypoxia–ischemia (HI) group and sham control group. All representative EEG epochs were acquired in the neonatal rats during quiet state to minimize the artifact. The overall voltage of EEG after left carotid artery ligation and before hypoxia **(A2)** showed a decreasing trend compared to the P3 baseline **(A1)**. During the HI induction **(A3)** and immediately after hypoxia **(A4)**, the voltage of EEG were significantly lower than baseline **(A1)**. The voltage gradually recovered at 24 h and 48 h after HI induction, but was still lower than that of P4 and P5 in the sham group, respectively **(B,C)**. The EEG voltage at 72 h, 96 h and 120 h after HI induction gradually returned to the level of the sham group at P6, P7 and P8, respectively, and the EEG wave was more continuous **(D–F)**. EEG was acquired in a P3 rat in the HI group during the baseline **(A1)**, after left carotid ligation, but before hypoxia **(A2)**, during hypoxia **(A3)**, immediately after HI **(A4)**, 24 h after HI **(B2)**, 48 h after HI **(C2)**, 72 h after HI **(D2)**, 96 h after HI **(E2)**, and 120 h after HI **(F2)**. In the sham group, EEG was acquired from P4 **(B1)**, P5 **(C1)**, P6 **(D1)**, P7 **(E1)**, and P8 **(F1)**. V, volts, S, seconds.

**FIGURE 6:**
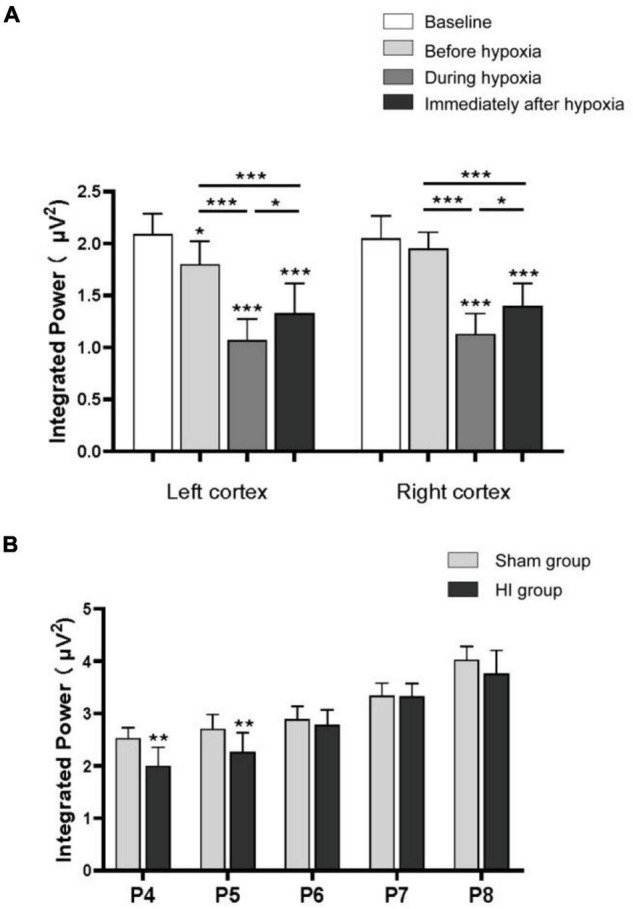
Integrated power of background electroencephalography (EEG). The integrated power analysis in the P3 rats of the hypoxia–ischemia (HI) group during the baseline, after left carotid ligation, but before hypoxia, during hypoxia, and immediately after HI **(A)**. The integrated power of the left cortex decreased compared with the baseline after the ligation of the left carotid artery. The power decreased significantly during hypoxia and immediately after hypoxia in both sides of the cortex. Integrated power value analysis from rat pups every day between P4 and P8 in HI and sham group **(B)**. The integrated power value returned to normal with the increase of time after hypoxia in the HI group compared with the sham group. All EEG epochs were acquired in the neonatal rats during quiet state to minimize the artifact. *p < 0.05; **p < 0.01; ***p < 0.001.

In very immature brain rats (P3), baseline EEG activity was discontinuous, with bursts of higher amplitude activity and long intermittent periods of voltage attenuation. After the removal of movement artifacts, some spikes and sharp waves were also observed (Figure 5A1). In the P3 rats of the HI group, there was a trend toward voltage suppression following left carotid artery ligation and before hypoxia (Figure 5A2). To further analyze the integrated EEG power of the background EEG activity, the left cortex integrated value after carotid artery ligation decreased compared with the baseline values (Figure 6A). During HI induction (Figure 5A3) and immediately after hypoxia (Figure 5A4), the upper and lower EEG margins in both cortices were significantly lower than those observed at baseline. Quantitative analyses revealed a significant reduction in the integrated EEG power during and immediately after hypoxia in both cortices as compared to baseline values (Figure 6A). However, no significant differences were observed between the left and right cortices (Figure 6A).

In the P4 of the sham group, EEG features were characteristic of discontinuous low-amplitude waves (Figure 5B). The EEG became more continuous with increased voltage as the age increased. The EEG of the HI groups still exhibited depressed voltage and less continuous values than did the sham group 24 h and 48 h after HI induction (Figures 5B,C). A quantitative integrated EEG power analysis showed that the voltage in the HI group of P4 and P5 was significantly lower than that in the sham group (Figure 6B). At 72 h, 96 h and 120 h after HI induction, the EEG voltage gradually returned to the level of sham group and the EEG wave was more continuous (Figures 5D–F). There were no significant differences in the integrated EEG power between P6 and P8 in both groups (Figure 6B).

## Discussion

In this study, electroclinical seizures and EGS that were related to changes in EEG and EMG activity were observed following HI induction in immature rats. Seizures in human neonates are characterized by brief, repeated, and subtle episodes (17–19). Although continuous video-EEG is the gold standard for seizure monitoring in human neonates with HIE (20), only one-third of neonatal EEG seizures display clinical signs on simultaneous video recordings (21). According to a previous study (22), EMG can be used to attain some level of discrimination between behaviors associated with seizure-like events in neonatal rats used to model HIE. Therefore, in this study, using video-EEG combined with EMG monitoring allowed us to accurately associate EEG waveforms with rat behavior and distinguish non-obvious electroclinical seizures.

Previous studies also reported tonic, clonic, and tonic–clonic seizures during hypoxia in P7 rats whose right carotid arteries had been ligated (23). The same authors demonstrated that, following HI induction, electroclinical seizures and EGS occurred spontaneously for 24 h in most rats, and no EGS were observed 48 h after HI (23). In our study, all pups experienced frequent EGS, and most of them developed frequent and long-duration electroclinical seizures during hypoxia. In human neonatal HIE, the first seizure has been observed 6–20 h after birth (24, 25). However, for some practical reasons, human EEG is usually performed many hours after birth; therefore, the actual onset of epilepsy may be earlier. Doctors often need to deal with life-threatening symptoms, such as dyspnea and acidosis, in very premature infants with perinatal hypoxia; therefore, they are unable to monitor EEG in the early postnatal period. In a study on human perinatal arterial ischemic stroke by Low et al., most seizure events were electrographic-only seizures 72 h after birth (26). In our study, half of the pups still experienced seizures 48 and 72 h after HI induction, and subclinical seizures continued to occur 120 h or more after birth, suggesting that even if there are no observable electroclinical seizures in the early stages of HI induction, EGS will still occur in rats in the following period. In the animal model of full-term neonatal HIE (P7), no seizures were found 72 h after hypoxic–ischemic injury (23). This is possible because more severe brain damage is seen in lesser-age HIE models. Consistent with our study, human preterm newborns are more prone to seizures, and outcomes are worse in preterm than in term newborns (27).

The current study emphasizes the significance of developmental assessment in neonatal rats as short-term neurofunctional outcomes have been shown to correlate with long-term functional deficits (28). Indeed, in human neonatal infants, the absence or persistence of neonatal reflexes is predictive of the extent of later functional impairments in patients with brain injury (29, 30). This is the first time that behavior is associated with EEG in the neonatal rat model of HI. In our study, we also confirmed the presence of poor neurofunctional outcomes in behavioral tests in rats with frequent seizures in the early stage.

In addition to seizures, the background voltage suppression of EEG was observed in HI neonatal rats. Background EEG was dominated by low-amplitude, mixed-frequency activity in the early developmental period (P3). In P4–P8 rats, longer periods of higher amplitude activity were observed with increasing age. A previous study reported that the EEG voltage in the cortex ipsilateral to the ligated carotid artery was lower than that in the contralateral cortex in HI rats (23). In human infants with HIE, a severely depressed EEG background is a reliable predictor of severe brain injury and abnormal long-term neurologic outcomes (16, 31). In our research, we also found that the left cortex integrated value after carotid artery ligation decreased compared with the baseline value. There was a visually obvious decrease in interictal EEG voltage during and immediately after hypoxia in both cortices. Similar results were also found in our neonatal rat model to simulate premature human infants with HIE. According to a study on neonatal HIE, an EEG amplitude still showing suppression and discontinuity 48 h after birth indicates a poor prognosis (31). In our animal model, the EEG of the HI groups still exhibited depressed voltage and was less continuous 24 and 48 h after HI induction, but it gradually recovered after 72 h.

In this study, we investigated the characteristics and evolution of clinical and subclinical seizures over a 120-h period in neonatal rats with HI. Due to technical limitations (e.g., small head size, unfixed skull, and the need for neonatal rats to live with their mothers for breastfeeding support) associated with recording from immature rodents, most electrophysiological studies on epileptiform activity in neonatal animals have been performed *in vitro* using brain slices (32), and only a few studies have used behavioral measures and older animals to evaluate neonatal seizures (22, 33). Overall, our findings on the HIE model in neonatal rats correlate with some features of epileptic seizures and EEG characteristics as compared with older rodent models and human infants in previous studies. However, this study has some limitations. The recording length was inadequate to acquire more details on epileptic seizures, as neonatal rat pups must live with their mothers for breastfeeding support and cannot be separated from the dam for a long time. We elected to record data once per day for each pup (P4–P8) to reduce the damage caused by inserting and unplugging electrodes. In a previous study involving P6 rat models (34), the authors used a novel wireless telemetry system that theoretically allows for continuous monitoring for up to 2 years without surgical reimplantation. In this way, EEG data can be recorded without inserting and unplugging electrodes in each recording session, and rats are allowed to move freely. However, the electrodes used in that study were still too large for the P2 rats used in the current study. The head circumference of neonatal rats increases faster, and long-term electrode embedding recording may limit the development of the head. Therefore, future studies should aim to develop smaller radio electrodes and record EEG data from multiple other brain regions to obtain more detailed information.

## Conclusion

This study is the first to report that aEEG and EMG activities can be recorded continuously for 5 days after hypoxic–ischemic induction in 3-day-old neonatal rats. This method replicates most seizure characteristics observed in preterm infants with perinatal arterial ischemic stroke and allows aEEG data to be examined in relation to short-term neurofunctional outcomes.

## Data Availability Statement

The raw data supporting the conclusions of this article will be made available by the authors, without undue reservation.

## Ethics Statement

The animal study was reviewed and approved by Beijing Friendship Hospital Ethics Committee, Capital Medical University.

## Author Contributions

XS contributed to the project conception and design, preformed laboratory work, data analysis, and wrote the manuscript. FX performed data analysis and supervision. JW and LG assisted with laboratory work. YL and QJ assisted with statistical and data analysis. LY and HC revised and approved the final manuscript. All authors contributed to the article and approved the submitted version.

## Conflict of Interest

The authors declare that the research was conducted in the absence of any commercial or financial relationships that could be construed as a potential conflict of interest.

## Publisher’s Note

All claims expressed in this article are solely those of the authors and do not necessarily represent those of their affiliated organizations, or those of the publisher, the editors and the reviewers. Any product that may be evaluated in this article, or claim that may be made by its manufacturer, is not guaranteed or endorsed by the publisher.
